# Acute heat-stress testing in coral bleaching research: a review of research using the Coral Bleaching Automated Stress System (CBASS)

**DOI:** 10.1007/s00338-026-02862-7

**Published:** 2026-04-04

**Authors:** Ilan E. Bubb, Kay Watty, Lyza Johnston, Verena Schoepf

**Affiliations:** 1 Department of Freshwater and Marine Ecology, Institute for Biodiversity and Ecosystem Dynamics, University of Amsterdam, Amsterdam, The Netherlands; 2 Johnston Applied Marine Sciences, Commonwealth of the Northern Mariana Islands, Saipan, USA

**Keywords:** CBASS, Climate change, Coral bleaching, Heat-stress experiment, Experimental design, Thermal stress assay, Review

## Abstract

As climate change intensifies, identifying heat-resistant corals is essential for advancing coral‐bleaching research and informing reef conservation and restoration. The Coral Bleaching Automated Stress System (CBASS) is designed to meet this need by conducting acute heat-stress assays within a standardized methodological framework. Given its affordability and short duration, CBASS use has increased since 2020, warranting a first review of its application. Here, we assess (1) the scope of CBASS research including geographic and species representation, (2) variation in experimental design, (3) the range of measured response variables, and (4) how CBASS-derived heat responses compare with those from longer-duration experiments and in situ surveys. We then synthesize these findings to identify advantages and limitations and suggest improvements. We show that CBASS studies exhibit taxonomic and depth biases similar to other coral-bleaching experiments. Most studies include adequate biological replication but insufficient tank replication. Temperature treatments were highly standardized, whereas rationales for selecting light levels were lacking. Frequent tissue sloughing and mortality in the highest temperature treatment underscore the need to re-evaluate the use of extreme temperatures. Chlorophyll fluorescence and associated dose–response metrics such as ED50 were the most commonly measured response variables, but standardization and reporting warrant improvement. Notably, in-depth review of the few studies comparing acute and longer-duration stress assays indicates that it remains unresolved whether CBASS-derived coral responses reliably predict coral performance under real-world heatwave scenarios. Consequently, we caution relying solely on acute stress assays to inform reef conservation and genotype selection for restoration planning until further validation is achieved.

**Supplementary Information:**

The online version contains supplementary material available at 10.1007/s00338-026-02862-7.

## Introduction

Marine heatwaves are increasing in both frequency and intensity as a result of anthropogenic climate change (Oliver et al. 2018; Lough et al. 2018), significantly impacting coral reef ecosystems globally (Hoegh-Guldberg et al. 2014; Hughes et al. 2017). Heat stress causes the symbiosis between the coral host and its endosymbiotic dinoflagellates (Symbiodiniaceae) to break down, leading corals to expel their symbionts in a process known as coral bleaching. Because these symbionts normally provide much of the daily energetic needs of the coral through photosynthesis, bleached corals are energetically starved, making them more susceptible to disease and mortality, as well as reduced growth, reproduction, and overall health (e.g., Brown 1997; Hoegh-Guldberg 1999; Omori et al. 2001). As the ocean warms, mass coral bleaching events are becoming more frequent (Hughes et al. 2018). The fourth documented, and to date most severe, Global Coral Bleaching Event has been devastating coral reefs worldwide since 2023 (Doherty et al. 2025; Raoult et al. 2025; Spady et al. 2026). This raises questions of how coral reefs may persist under intensifying climate change and which coral species, populations, and genotypes are most likely to resist and survive heat stress; issues with significant implications for coral reef conservation, management, and restoration (e.g., Morikawa et al. 2019; Caruso et al. 2021; Colton et al. 2022).

Despite the increased frequency and severity of mass coral bleaching events, some coral species, populations, and genotypes demonstrate relative resistance to bleaching (e.g., Fine et al. 2013; Palumbi et al. 2014; Thomas et al. 2022), particularly those exposed to consistently warmer or highly variable environments (e.g., Coles and Riegl 2013; Safaie et al. 2018), indicating a potential resilience to climate change (Carilli et al. 2012; Schoepf et al. 2015; Rivest et al. 2017; Camp et al. 2019; Brown et al. 2024). These resistant corals have become key targets for coral reef conservation and restoration efforts (e.g., Caruso et al. 2021). However, identifying resistant corals before a bleaching event occurs remains challenging as the variation in thermal tolerance among coral species, populations, and even individual colonies are difficult to quantify under non-stress conditions (e.g., Rowan et al. 1997; Palumbi et al. 2014). Furthermore, controlled heat stress experiments simulating natural marine heatwaves are logistically challenging, time-consuming, and often lack standardization, limiting large-scale comparability (McLachlan et al. 2020; Grottoli et al. 2021).

Thermal tolerance is inherently time and magnitude dependent, with tolerated temperatures decreasing as exposure duration increases and vice-versa (Rezende et al. 2014). Environmental selection is thus expected to shape a trade-off between tolerance to acute versus chronic thermal stress, such that organisms may specialize in surviving extreme temperatures for short periods (e.g., highly variable environments such as tidepools) or enduring moderate, yet still stressful, temperatures over longer durations (e.g., mild heat stress event), but importantly not both (Rezende et al. 2014). This theory of trait selection is particularly relevant for coral thermal stress experiments aimed at identifying climate resilient coral species, populations or genotypes, as heat stress assays vary widely in duration and temperature, ranging from chronic, lower-temperature treatments lasting weeks or months to acute, high-temperature exposures lasting from hours to days (Grottoli et al. 2021). Moderate- and long-term experiments spanning seven or more days are often able to mimic natural heat stress events by targeting degree-heating weeks (e.g. Humanes et al. 2022), which describe the amount of time spent over historic average temperatures (Gleeson 1994), and are generally seen as ecologically relevant and representative of real-world responses to thermal stress (Grottoli et al. 2021). Acute and short-term experiments instead often rely on high treatment temperatures modeled off degree-heating days or even hours (e.g. Klepac et al. 2024) and do not mimic most natural heating events, except maybe those found in shallow reef flats due to low tides (Grottoli et al. 2021). A crucial question is therefore whether acute heat stress assays can predict coral responses to natural marine heatwaves as ecological theory suggests that the correlation between acute and chronic stress tolerance is likely low (Rezende et al 2014).

The Coral Bleaching Automated Stress System (CBASS; see Box 1) is one of these acute stress protocols designed to conduct rapid assessments to identify corals with relatively high bleaching resistance (Voolstra et al. 2020; Evensen et al. 2023b). CBASS is a relatively affordable and portable experimental system that was developed to simplify and standardize short-term acute thermal stress assays used previously in other studies (e.g. Palumbi et al. 2014; Morikawa and Palumbi 2019). Its temperature profiles follow a rapid heat-pulse with multiple, standardized temperature levels and durations (Voolstra et al. 2020; Evensen et al. 2023b). However, while acute stress assays are defined as lasting up to 7 days (Grottoli et al. 2021), CBASS can be completed between seven and 18 h. The short duration of CBASS assays could thus be a key advantage in terms of logistics and costs if coral responses to acute heat stress reflect outcomes under chronic exposure and real-world marine heatwaves (e.g., Naugle et al. 2025). However, it remains unclear how frameworks aimed at increasing comparability among coral heat stress experiments (Grottoli et al. 2021; McLachlan et al. 2020; Voolstra et al. 2024) are being applied by the diverse and rapidly growing CBASS user groups and whether variation in experimental design and underreporting of critical information persist (McLachlan et al. 2020). Furthermore, while some aspects of CBASS experimental design, such as temperature profiles, are standardized, this is not the case for other important variables (e.g., light intensity, water quality and flow rate, fragment size, collection procedures, etc.), which can be problematic as they have been shown to affect heat tolerance measurements (Nielsen et al. 2022).

Crucially, the extent to which acute heat-stress assays such as CBASS can predict real-world bleaching outcomes remains unclear. Recent work shows that corals respond to acute and chronic heat stress through independent genetic and molecular pathways (Savary et al. 2021; Humanes et al. 2024), supporting the ecological theory outlined above, while other studies find some comparable responses between acute stress assays and longer thermal stress experiments (Voolstra et al. 2020; Evensen et al. 2021; Cunning et al. 2024). Thus far, only two studies have compared CBASS responses to survival under a natural marine heatwave (Naugle et al. 2025; McRae et al. 2024), with only one (Naugle et al. 2025) directly comparing the same colonies in both CBASS and in situ. Both studies show mixed results regarding CBASS’s ability to predict surviving genotypes (McRae et al. 2024; Naugle et al. 2025). Given CBASS’s growing popularity, it is therefore timely to conduct a review to address these knowledge gaps and improve methodologies for future work using CBASS. To achieve this, the review was approached with four specific goals:

1. To report the current application of CBASS in research, including the geographic distribution of studies, the diversity of species used, and collection details.
2. To synthesize data on the standardization and reporting of experimental design choices and parameters to assess comparability of results.
3. To evaluate the response variables measured across studies and their utility in terms of assessing bleaching resistance.
4. To analyze the subsection of literature that experimentally compares CBASS acute stress assays with longer-term (hereafter referred to as “classic”) heat stress tests and in situ data regarding experimental design, response variables, and correlation of responses, to determine the ecological relevance of CBASS. Here, we define longer-term to refer to assays that last longer than seven days, corresponding to medium- or long-term assay classifications under the Grottoli et al. (2021) framework. Overall, this review serves to provide a better understanding of advantages and limitations of CBASS in its current state and provide CBASS-specific recommendations to help ensure reliable comparisons across studies and enhance reproducibility, ultimately improving global assessments of coral thermal tolerance in an appropriate ecological context.

**Box 1:** Coral Bleaching Automated Stress System (CBASS)

CBASS was introduced in 2020 (Voolstra et al. 2020). It aims to provide a standardized, short-term acute heat stress assay to quickly assess coral thermal thresholds. The CBASS approach consists of 18-h thermal profiles designed to mimic shallow water temperature profiles. However, note that CBASS uses higher-level temperature treatments that often far exceed those found in most reef settings. Each profile includes a 3-h ramp-up, 3-h heat-hold, 1-h ramp-down, and overnight reprieve period. Treatments are based on the local maximum monthly mean (MMM) as the control temperature, with increments corresponding to MMM + 3 °C, + 6 °C, and + 9 °C to elicit a broad bleaching response To determine thermal thresholds, the tolerance metric Effective Dose 50 (ED50) is calculated from measurements of, for example, photosynthetic efficiency (Fv/Fm*). Here, Fv/Fm represents the maximum quantum yield of photosystem II, and the asterisk denotes the point in the assay at which photosynthetic efficiency is typically measured. ED50 describes the temperature at which the measured response variable reaches 50% of its initial value and is modeled using log-logistic regression, i.e., dose–response curves (Voolstra et al. 2024; simulated example below)	

## Methods

### Literature search

The literature reviewed included studies that conducted heat-stress experiments using the Coral Bleaching Automated Stress System (CBASS; Voolstra et al. 2020; Evensen et al. 2023b) or other acute heat-stress assays that were primarily based on the same experimental design. The literature search was conducted using Google Scholar, and Web of Science. The search began in January 2025 and was completed on March 19, 2025. Search terms included ‘Coral Bleaching Automated Stress System’, ‘CBASS coral bleaching’, ‘coral thermotolerance’, ‘coral thermal thresholds,’ and variations thereof. In addition to keyword searches, a forward citation search of Voolstra et al. (2020) was conducted in Google Scholar, using the ‘Cited by’ feature. Studies that did not specifically mention CBASS were included if they conducted a short-term heat-stress assay within a single day, using a heat-pulse profile with multiple temperature levels designed to induce a broad bleaching response to assess relative coral thermal tolerance (e.g., Marzonie et al. 2022). At the time of writing, 25 publications, two dissertations, and two preprints met the criteria and were included in this review, for a total of 29 studies. Although the preprints have not yet been peer-reviewed at the time of writing, they were included because their experimental decisions remain unchanged. In some cases, multiple publications reported on the same or similar CBASS experiments, while others presented aggregated results from several CBASS experiments. When experiments were explicitly distinct, i.e., performed independently, they were treated as separate experiments in terms of experimental replication. However, design choices were considered at the publication level to evaluate the reporting behavior across studies.

### Data collection

The data collection process was modeled on previous literature reviews that synthesized a substantial number of coral heat-stress experiments with the aim to increase comparability among coral bleaching experiments by providing a framework for best practice recommendations (McLachlan et al. 2020; Grottoli et al. 2021). Data were grouped into subsections to address the respective goals: (1) Current application, geographical distribution, taxonomic and collection details, (2) Experimental design and parameter reporting, (3) Measured response variables and associated metrics, (4) Comparison of CBASS vs classic heat stress experiments.

#### Goal 1: Current application, geographical distribution, taxonomic and collection details

Metadata collected included the publication year, author affiliation(s), the geographical location of collection sites, coral species and morphologies assessed, collection depth, and whether tested corals were wild or cultured specimens (either from in- or ex-situ nurseries). To assess geographical representation, local collection sites were grouped into the following geographical regions: Florida Reef Tract, Gulf of Aqaba, Central Red Sea, Southern Red Sea, Gulf of Aden, Northern Great Barrier Reef, Central Great Barrier Reef, Southern Great Barrier Reef, Coral Sea (separate from Great Barrier Reef), American Samoa, French Polynesia, Southern Taiwan, Palau, and Hawaii. Coral morphologies were classified broadly as branching, hemispherical, corymbose, tabular, encrusting, or digitate, following commonly used coral morphological classifications (Pratchett et al. 2015; Cresswell et al. 2020).

#### Goal 2: Experimental design and parameter reporting

**Fig. 1:**
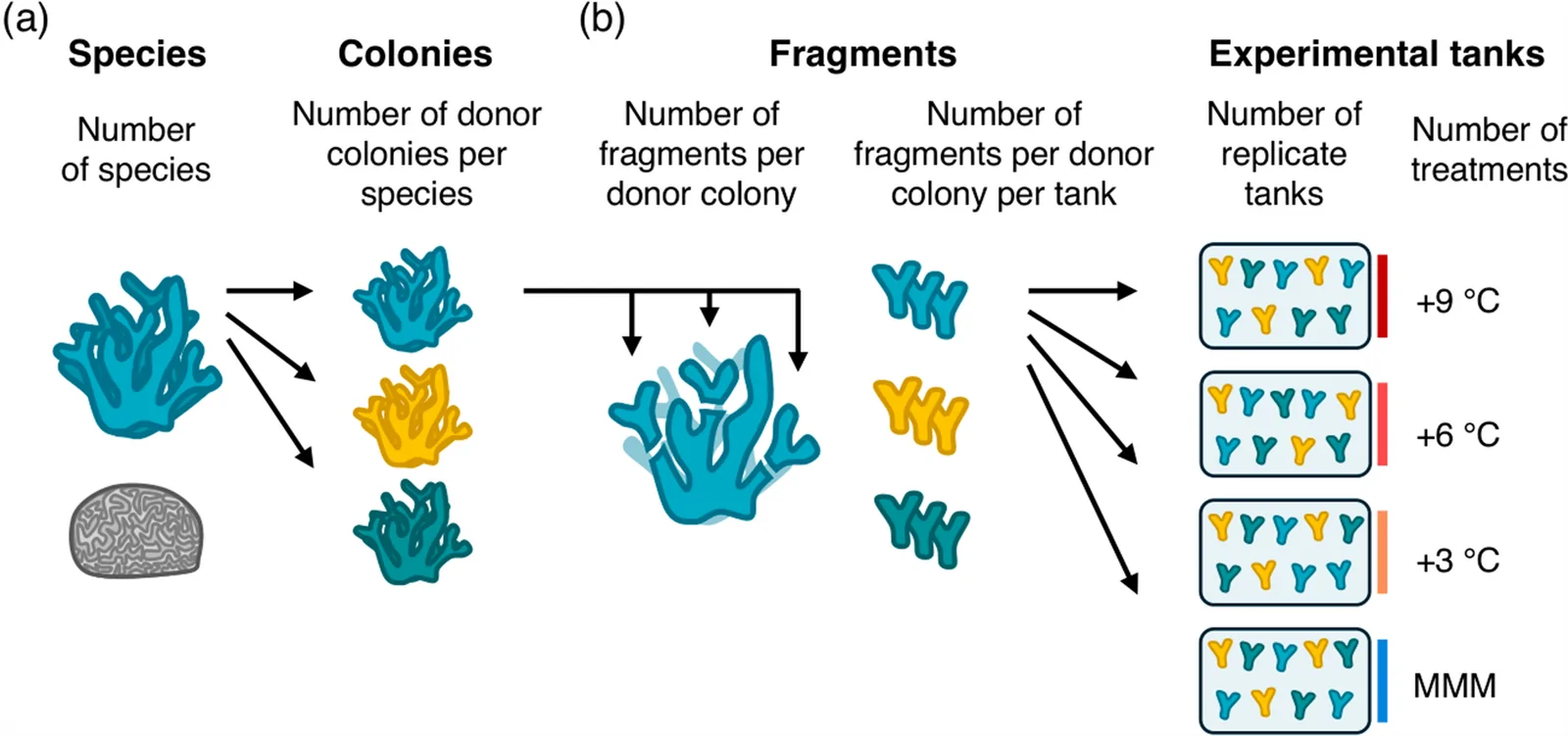
Flowchart illustrating a generalized overview of CBASS experimental sampling and replication, inspired by McLachlan et al. (2021) and Voolstra et al. (2020). **a** Coral sampling design prior to CBASS experiments. **b** Donor colony fragmentation for biological replication and experimental replication. MMM, maximum monthly mean temperature

Data on experimental design were grouped into experimental sampling and replication, experimental temperature and light regimes, and other reported relevant abiotic parameters. Replication data included the number of unique genotypes or colonies, and the number of replicate fragments per colony per treatment, and the number of replicate tanks per temperature treatment. Terminology related to coral sampling and experimental replication follows the outline illustrated in Fig. 1. CBASS design data contained the chosen baseline (control) and treatment temperatures, temperature ramping information over the experimental duration, light intensity during the experiment, and the justifications for each of these design choices when provided. Other parameters assessed included water source (natural vs. artificial) and quality (salinity, dissolved oxygen, nutrients, pH, and other carbonate chemistry parameters), flow rate, fragment size and collection procedures, including whether corals had an acclimation and/or recovery period prior to testing.

#### Goal 3: Measured response variables and associated metrics

Measured coral response variables and associated metrics across CBASS experiments were identified and grouped into the following categories (adapted from McLachlan et al. 2020): photosynthetic capacity, bleaching phenotype, holobiont phenotype, and holobiont genetic composition (including symbiont and microbiome identification). For the most commonly measured variable, maximum quantum yield of photosystem II (Fv/Fm), standardization was assessed based on reported information on methodological considerations of Pulse-Amplitude-Modulated (PAM) fluorometry, including the instrument model, dark-acclimation time, and instrument settings. Consistency of response variables was also recorded for studies that did repeat CBASS assays to access reproducibility of results.

#### Goal 4: Comparison of CBASS vs classic heat stress experiments and in situ heatwaves

We further filtered the identified literature to find all studies that represented method assessments comparing the ability of CBASS to derive similar conclusions regarding thermal tolerance as classic experiments. Here, classic experiments refer to longer-term heat-stress assays intended to be more representative of natural marine heatwaves and thus used for comparison with CBASS assays. Overall, seven studies in the review reported results on both CBASS and classic experiments. Of these, three were excluded as they were not assessing the comparability of CBASS and classic experiments. The other four studies included here were method assessments whose primary goal was to compare CBASS and classic experiments in order to evaluate the potential for CBASS as a rapid screening tool (Voolstra et al. 2020; Evensen et al. 2021; Cunning et al. 2024; Klepac et al. 2024).

Key design considerations of the classic experiments were recorded, including light levels, duration of the test, temperature treatments, whether comparisons were made at a genotypic or population level, and measured response variables. For each response variable related to heat tolerance, we assessed whether CBASS and classic experiments resolved similar rankings of thermal tolerance at both the population and genotypic level, as indicated by a statistically significant positive correlation. In other words, we tested whether the populations or individuals identified as most or least resistant to thermal stress were the same for each methodology. However, because Evensen et al. (2021) only compared results from a single population, agreement between CBASS and classic experiment is instead reported based on whether the variables showed a lack of significant differences between experiments.

Given the popularity of Fv/Fm-derived metrics in CBASS, such as ED50 and ΔFv/Fm, and the assertion that they are particularly well-suited for acute stress assays (Voolstra et al. 2020), we also reviewed whether these metrics correlated with, or resolved the same patterns as, other variables in both CBASS and classic experiments. Here, agreement at the individual level was defined as a significant correlation in the same direction between the CBASS Fv/Fm metric and other variables. At the population level, agreement was again based on whether CBASS Fv/Fm metrics resolved the same ranking as other response variables. In other words, similar to above, we tested whether the populations or individuals identified as most or least resistant to thermal stress according to CBASS Fv/Fm were the same for each response variable across both methods. As above, for the Evensen et al. (2021) paper, agreement was reported based on whether the other response variables showed the same pattern as the CBASS Fv/Fm variable.

Lastly, in situ comparisons with CBASS data were recorded and qualitatively ranked to describe whether the in situ responses agreed with CBASS results. Comparisons came from either previous literature that studied the same system, species and/or populations that were used in CBASS experiments, or from papers that conducted CBASS assays and in situ monitoring within the same study.

## Results

### Goal 1: Current application in research, geographical distribution, taxonomic and collection details

**Fig. 2:**
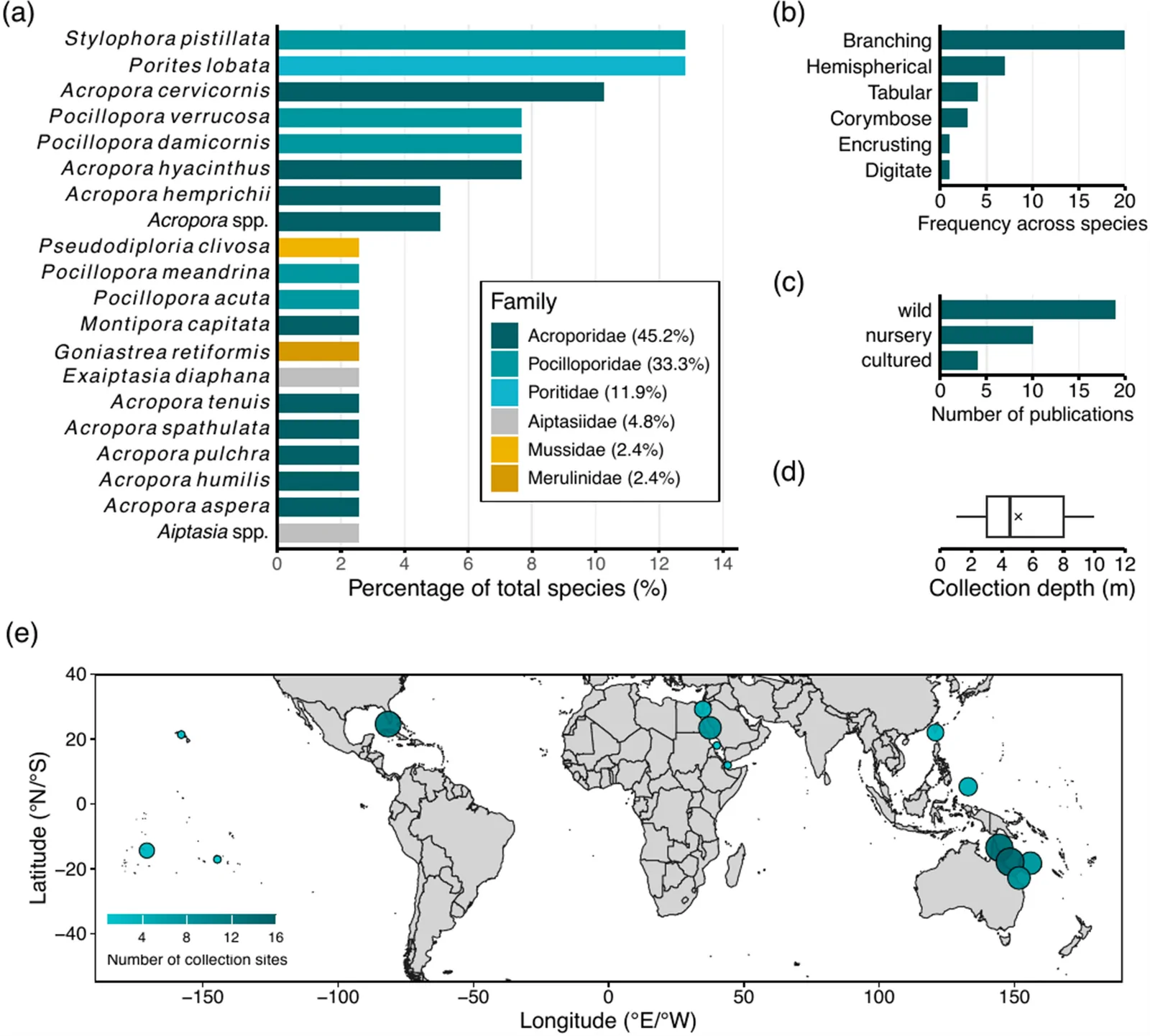
Taxonomic, morphological and site representation of corals used in CBASS assays. **a** Relative abundance of scleractinian coral and anemone species used in CBASS experiments as the percentage of total species used. The legend shows the percentages per color coded families. **b** Morphology frequencies of scleractinian corals across species. **c** Numbers of specimen origins coming from either wild habitats, nurseries or cultures across publications. **d** Depth range from which wild or in situ nursery corals were collected. The solid line and ‘x’ depicts the median and mean collection depths, respectively. **e** Global distribution of coral collection sites from wild and nursery corals used in CBASS experiments. The color gradient represents the number of collection sites within the broader geographical regions, while circle sizes are proportional to the frequency of collections, highlighting hotspots. Indicated broad geographical locations on the map, from west to east, are American Samoa, Hawaii, French Polynesia, Florida Reef Tract, Gulf of Aqaba, Central Red Sea, Southern Red Sea, Gulf of Aden, Southern Taiwan, Palau, Northern Great Barrier Reef, Central Great Barrier Reef, Southern Great Barrier Reef, and Coral Sea (Marine Park)

There was a sharp increase in publications per year in 2021 following the initial introduction of CBASS in 2020 (Voolstra et al. 2020), and again in 2024 following the publication of the comprehensive CBASS methodological framework (Evensen et al. 2023b). To date, 90.0% of CBASS publications were authored by researchers affiliated with academic institutions. The other ten percent were conducted at independent conservation and research organizations, such as zoos or aquariums. Most studies were conducted on scleractinian corals (N = 27), with a few exceptions using anemones (Actiniaria; N = 2) (Fig. 2a). The most commonly studied taxonomic families were Acroporidae (45.2%) and Pocilloporidae (33.3%). While different morphologies were represented among the scleractinian corals, branching species dominated (57.9%) (Fig. 2b). Most collected corals were wild types, followed by corals originating from nurseries (Fig. 2c). To minimize the likelihood of sampling clonal genotypes, colonies were often collected 3–10 m apart. Among studies using wild corals, 21.1% were genetically confirmed genotypes, while 57.9% relied on colony distance as a proxy. In contrast, nursery and cultured corals typically had known genotypes from prior genetic analyses. Four studies conducted CBASS experiments on cultured specimens of which half included the anemones. Seventy-six percent of studies that used wild or nursery corals reported the collection depth. Corals were collected between 0 and 16.4 m and on average from 5 m across reefs globally (Fig. 2d). The date of coral collection was reported in 86.2% of studies, and the date of the CBASS experiment in 62.1%. Collection site hotspots were identified in the Central Great Barrier Reef (n = 16 sites), Northern Great Barrier Reef (n = 15), and Florida Reef Tract (n = 13), followed by the Central Red Sea (n = 9), Southern Great Barrier Reef (n = 9), and Coral Sea (n = 9) (Fig. 2e). Additionally, CBASS experiments were conducted on corals from reefs in Palau (n = 5), the Gulf of Aqaba (n = 4), Southern Taiwan (n = 4), American Samoa (n = 3), the Gulf of Aden (n = 1), French Polynesia (n = 1), the Southern Red Sea (n = 1), and Hawaii (n = 1).

### Goal 2: Experimental design and parameter reporting

#### Experimental sampling and replication

Sixty-nine percent (N = 20) of studies assayed one species, 20.4% (N = 6) included two species, and three studies (10.3%) tested three to four species. When a single species was studied, the focus was often on variations in heat tolerance across spatial scales (e.g., Naugle et al. 2024) and/or genotypes (Cunning et al. 2021). In contrast, studies that included multiple species often investigated species-level variation in thermal tolerance (e.g., Evensen et al. 2022). The number of colonies per species sampled ranged from 1–60 colonies per assay with a median of 9.5 (mean = 14.8). Between 1–10 fragments per donor colony per temperature treatment were distributed across replicate tanks, with one fragment being the most common choice (66.7%). Fragment size was reported in 55.2% (N = 16) of studies, with length being the most common measurement, followed by surface area. One study reported fragment size in volume. Where data were available, experimental fragments had an average length of 3.9 ± 2.1 cm and an average surface area of 5.9 ± 3.5 cm^2^ (mean ± SD). Studies used one (N = 18; 62.1%), two (N = 5; 17.2%), or three (N = 6; 20.7%) replicate tanks per temperature treatment. Corals underwent an acclimation period before the CBASS experiment in 34.5% of studies, ranging from 30 min to two years. The acclimation period aimed to prime corals for experimental conditions upon transplantation. After fragmentation, 31% of studies included a recovery period, which varied from 3 h to 30 days.

#### Experimental temperature and light regimes

**Fig. 3:**
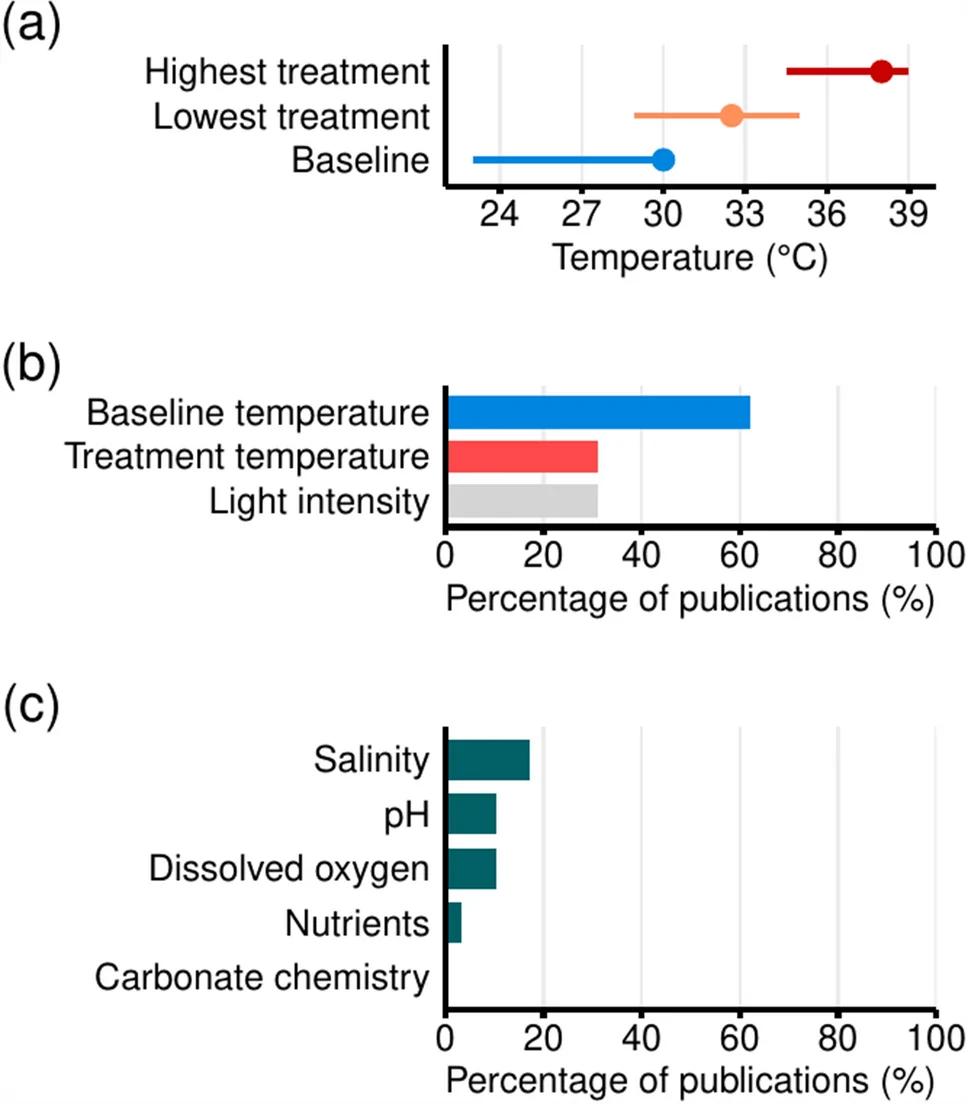
Temperature treatments used in CBASS, and proportion of studies that report temperature and light setting justifications and seawater parameters. **a** Temperature ranges used across CBASS experiments for baseline, lowest treatment, and maximum treatment temperatures. Points represent the median of the temperature ranges. **b** Proportion of studies that justified baseline/treatment temperatures and light intensities during assays. **c** Proportion of studies that reported seawater parameters across publications; carbonate chemistry refers to parameters other than pH, such as total alkalinity

Most experiments included four temperature treatments, including the control (68.9%), followed by eight treatments, at 13.7%, two treatments, at 10.3% and three or five treatments for the other 7.1%. Baseline temperatures ranged from 23 to 32 °C (median = 30 °C), lowest temperature treatments ranged from 28.9 to 35 °C (median = 32.5 °C), and highest temperature treatments ranged from 34.5 to 39 °C (median = 38 °C) (Fig. 3a). Temperature ramping was similar across experiments, with all studies using a 3-h ramp-up duration and a 3-h temperature hold. Ramp-down duration was mostly consistent, with 79.3% (N = 23) of studies using a 1-h ramp-down and 13.8% (N = 4) using 1.5–2 h. Only two studies (6.9%) had a substantially longer ramp-down of 16 h. While 62.1% of studies justified the chosen baseline temperatures, only about a third (31.0%) justified the treatment temperatures used during the experiment (Fig. 3b). Baseline temperatures were determined using either the MMM or ambient conditions, while treatment temperatures were selected based on Voolstra et al. (2020), pilot studies, or to induce a broad bleaching response. Light levels were justified either by in situ measurements, or based on long-term culturing conditions. When reported (55.2% of studies), timing of lights was highly standardized with all studies starting CBASS assays with lights turned on and turning them off before the ramp down. Light intensities ranged from 22–1600 μmol photons m^−2^ s^−1^ across studies with an average of 414 ± 302 μmol photons m^−2^ s^−1^ and a median of 325 μmol photons m^−2^ s^−1^. Sixty-eight percent of studies did not report justifications for their chosen light intensity. Only a single study did not report light intensities (Savary et al. 2021), but used the same experimental setup as Evensen et al. 2021.

#### Flow and seawater parameters

The majority (82.8%) of studies used a flow-through system, with 75.9% utilizing natural seawater. However, seawater chemistry parameters such as salinity (17.2%), pH (10.3%), dissolved oxygen (10.3%), nutrients (3.4%), or other parameters related to carbonate chemistry such as total alkalinity (0%) were rarely reported across studies (Fig. 3c). Additionally, seawater filtration was reported in only 27.6% of studies. Flow rates were reported in 72.4% of studies and ranged from 2 to 55 L h^−1^, with a median of 7.2 L h^−1^ and an average of 14.16 ± 16.96 L h^−1^.

### Goal 3: Measured response variables and comparability

#### Photosynthetic capacity

**Fig. 4:**
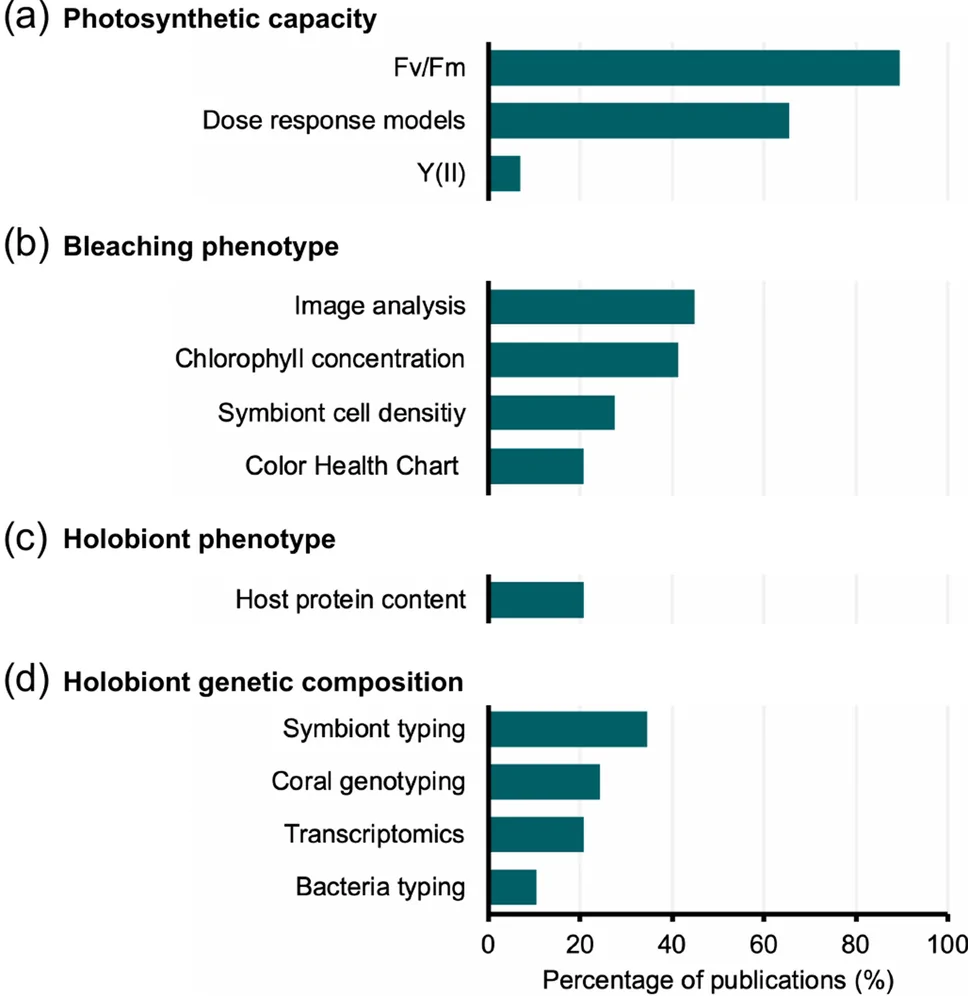
Percentage of publications which conducted CBASS experiments and measured response variables and other metrics listed in the following categories: **a** Photosynthetic capacity, **b** Bleaching phenotype, **c** Holobiont phenotype, and **d** Holobiont genetic composition, adapted from McLachlan et al. (2020). Note, Fv/Fm and Y(II) represent the maximum (dark-adapted) and effective (light-adapted) quantum yield of photosystem II, respectively. Symbiont typing, coral genotyping and bacteria typing were generally not used as a response variable, but were used to inform on the results of the study

The most common response variable measured in CBASS experiments was maximum photosynthetic efficiency (Fv/Fm), reported in 89.7% of publications (Fig. 4). Two studies (6.9%) also measured light-adapted effective quantum yield (Y(II)). These measurements, based on chlorophyll fluorescence, were most commonly taken using the Diving PAM (38.5%) or the Imaging PAM (23.1%), both from Walz GmbH (Effeltrich, Germany). Other instruments used included the Diving PAM II (11.5%), Junior PAM (11.5%), and both the Mini PAM (3.8%) and Mini PAM II (11.5%), all from Walz. Fv/Fm measurements were taken at a median of 7 h after the temperature ramp-up, typically between 19:00 and 22:00 in studies that reported the time of day (61.5%). Corals were dark-adapted before measuring, most commonly for 60 min (50% of cases), followed by 30 min (23.1%). When a fiber-optic probe was used, 1–3 different spots were measured per coral fragment (reported in 44.8% of cases). However, only five studies specified the fiber-optic distance to the coral fragment, which ranged from 2–10 mm. Fifty-eight percent of studies that measured chlorophyll fluorescence reported the PAM settings. Out of the 16 studies that reported settings, the most common parameters were Measuring Light Intensity (87.5%) and Saturation Pulse Intensity (87.5%), followed by the Gain (81.3%) and Damping (75%). Other reported settings included the Saturation Pulse Width (75%), the Measuring Light Frequency (25%) and the minimum fluorescence (F0) threshold before measuring (43.8%). F0 describes the baseline fluorescence when all photosystem II reaction centers are open, meaning minimal photochemical quenching is occurring. The values for Gain, Damping and Saturation Pulse Width were rather consistent with medians of 2 (dimensionless), 2 (dimensionless), and 0.8 s, respectively. Measuring light Intensity (1–12), Measuring Light Frequency (1–14), and Saturation Pulse Intensity (1–12) turned out to be more variable. F0 thresholds ranged from 100–500 mV for available data. Sixty-four percent of studies used Fv/Fm data in dose–response models using a three parameter log-logit function (reported in 55.6% of studies), of which, all but one derived the Effective Dose 50 (ED50). Two studies modeled the temperature at which the response variable decreased by 25%, which is the ED25.

#### Bleaching phenotype

Chlorophyll concentrations were quantified post CBASS experiments in 41.4% of publications, specifically measuring chlorophyll *a*. In four studies, chlorophyll *c*_2_ was additionally measured and total chlorophyll concentrations were reported instead. Bleaching was also quantified through image analysis (44.8%), either using software like ImageJ and MATLAB or relying on human interpretation to analyze pictures. In 20.7% of studies, the Coral Health Chart was used to quantify bleaching, a standardized color chart designed for in situ assessment of coral health (Siebeck et al. 2006). Cell densities of the dinoflagellate symbionts were quantified in 27.6% of studies. In two recent publications, the Normalized Difference Vegetation Index (NDVI) was calculated as a proxy for chlorophyll *a* (Naugle et al. 2024; Denis et al. 2024).

#### Holobiont phenotype

Across studies, 20.7% measured host protein content to assess energy reserves and the metabolic health of the host. Measurements were taken post CBASS and additionally before the experiment in one study. Individual studies also quantified catalase activity (Nielsen et al. 2022), hydrogen peroxide (Schlotheuber et al. 2024), or reactive oxygen species (Lane 2023). Two studies modeled coral mortality (Naugle et al. 2021) or quantified rapid tissue loss (DeMerlis 2024). Another two studies conducted respirometry, measuring photosynthesis and respiration (Evensen et al. 2021, 2023a).

#### Holobiont genetic composition

Symbiodiniaceae were identified to at least the clade or genus level in 34.5% of studies, primarily using ITS2 sequencing (80%), and corals were genotyped in 24.1%. Transcriptomics were conducted to examine gene expression in CBASS experiments (20.7%). Additionally, bacterial composition was analyzed in 10.3% of studies.

#### Reproducibility

Three studies tested the same coral genotypes twice to assess the reproducibility of CBASS results (Cunning et al. 2021, 2024; Naugle et al. 2025). The second assay was repeated between four months and up to two years after the first. Both Cunning et al. (2021; 2024) found that the ED50 between the two assays were significantly and positively correlated, but could shift by 0.51 °C on average between sampling times (Cunning et al. 2021). NDVI ED50 and Retained NDVI were also significantly correlated between time periods in Naugle et al. (2025). Unlike in the Cunning et al. papers, Fv/Fm ED50, ΔFv/Fm and ΔFv/Fm Width all lacked significant correlations between the repeated assays (Naugle et al. 2025).

### Goal 4: Comparison of CBASS with classic heat stress experiments and in situ heatwaves

#### Experimental design

All four studies comparing CBASS with heat stress experiments lasting seven or more days (i.e. classic) focused on a single species of coral, with two using *Stylophora pistillata* (Voolstra et al. 2020; Evensen et al. 2021), one using *Acropora pulchra* (Cunning et al. 2024), and the last one *Acropora cervicornis* (Klepac et al. 2024). Three studies compared each methodology’s ability to resolve similar rankings in heat resistance among individuals coming from nursery environments (Klepac et al. 2024; Voolstra et al. 2020; Cunning et al. 2024), two studies compared each methodology’s ability to resolve differences in heat tolerance between populations coming from two different environments (Klepac et al. 2024; Voolstra et al. 2020), and one study compared each methodology’s ability to resolve similar heat stress responses from a single population (Evensen et al. 2021) (Table 1). We also evaluated agreement between CBASS-derived Fv/Fm metrics and other response variables in both CBASS and classic experiments (Table 2).

**Table 1:** Overview of studies comparing CBASS and classic experimental methods. The agreement (✓) and disagreement (X) between methods are shown for the respective response variables per study. Blank cells indicate which variables were not assessed in the respective study. T, temperature; PAR, photosynthetically active radiation; BST, bleaching stress temperature. Klepac et al. (2024) and Voolstra et al. (2020) compared experiments using a specific CBASS treatment (34.3 °C and 36 °C, respectively)

Variable	Klepac et al. (2024)	Voolstra et al. (2020)	Evensen et al. (2021)	Cunning et al. (2024)
Classic Experiment Duration (days at BST)	Long (56 Days)	Moderate (8 Days)	Moderate (7–11 Days)	Moderate (8 days)
ΔT between max treatment in CBASS and Classic experiments	5 °C	4 °C	0 °C	5 °C
ΔT of compared CBASS and Classic treatments	2.8 °C	1 °C	0 °C	5 °C
ΔPAR between CBASS and Classic experiments	122 µmol lower in CBASS	418 µmol higher in CBASS	Same	Same
*Comparison at individual level*				
Fv/Fm metrics	X	✓		✓
Chlorophyll concentration	X	X		
Host protein concentration	X	X		
Symbiont density		X		
Pigment change		X		
*Comparison at population level*				
Fv/Fm metrics	X	✓	✓	
Chlorophyll concentration	X	X	✓	
Host protein concentration	X	X	✓	
Symbiont density		✓	X	
Pigment change		✓		
Respiration rates			X	

Classic experiments are classified in terms of duration based on Grottoli et al. (2021)

**Table 2:** Overview of methods comparison studies and whether CBASS-derived Fv/Fm metrics correlated with or resolved the same patterns as other variables in both CBASS and classic experiments. Blank cells indicate variables not assessed in the respective study

Variable	Klepac et al. (2024)		Voolstra et al. (2020)		Evensen et al. (2021)		Cunning et al. (2024)	
Method	CBASS	Classic	CBASS	Classic	Combined		CBASS	Classic
*Comparison at individual level*								
Fv/Fm metrics		X		✓				
Chlorophyll concentration	X	X	X	X				
Host protein concentration	X	X	X	X				
Symbiont density			*	*				
Pigment change			X	X				
Respiration rates								
*Comparison at population level*								
Fv/Fm metrics		X		✓	✓			✓
Chlorophyll concentration	X	X	✓	X	✓			
Host protein concentration	X	X	✓	X	✓			
Symbiont density			X	X	X			
Pigment change			X	X	X			

^*^Denotes that original data was not provided in repository

**Fig. 5:**
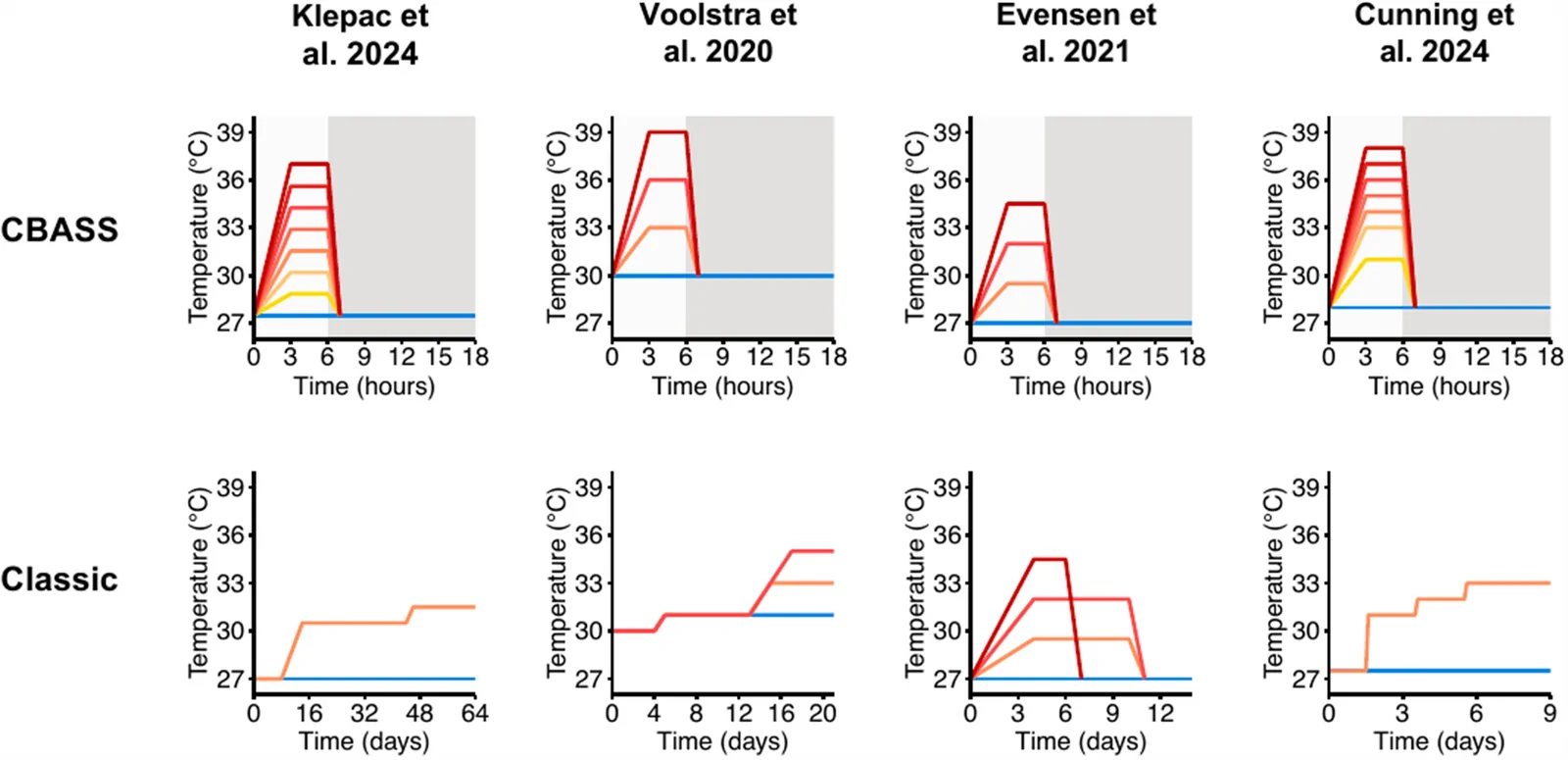
Comparison of CBASS and classic experimental designs across method assessment studies. CBASS experiments were conducted over 18 h, while classic experiments lasted upwards of 64 days. Blue lines indicate the control conditions, and other lines represent the respective heating treatments. Figures have been adapted from the original publications (Voolstra et al. 2020; Evensen et al. 2021; Cunning et al. 2024; Klepac et al. 2024)

Experimental design of the four method assessments differed across studies (Fig. 5, Table 1). Duration of the classic experiments varied from lasting two months to as short as 7 days. Despite referring to these classic experiment comparisons as “long”, “longer” or “chronic” experiments, the Grottoli et al. (2021) framework only classifies the two-month long experiment as a long-term heat stress experiment and the other three as marginally moderate-term assays. Three of the studies used stepwise temperature ramping to a maximum temperature that was lower than the CBASS maximum, whereas one study (Evensen et al. 2021) used a continual temperature ramp with both ramp-up and ramp-down phases, reaching the same maximum temperature as the CBASS assay (Fig. 5, Table 1). However, on average, CBASS experiments reached a maximum temperature that was 3.5 ± 2.06 °C (mean ± SD) higher than their classic experiment comparisons. Light settings also often varied between CBASS and classic experiments (Table 1). Evensen et al. (2021) and Cunning et al. (2024) used the same light levels, while Voolstra et al. (2020) and Klepac et al. (2024) used different light levels. Notably, Voolstra et al. (2020) used much higher light levels in CBASS (600 μmol photons m^−2^ s^−1^) than in the classic experiment (182.5 μmol photons m^−2^ s^−1^).

#### Agreement between responses to short-term vs longer-term experimental heat stress

Three of the four studies found strong correlations between Fv/Fm-derived metrics from CBASS and classic heat stress experiments (Table 1), reporting that these metrics consistently identified similar populations and individuals with higher or lower resistance to thermal stress. However, Klepac et al. (2024) found no positive correlation between the two methods for Fv/Fm-derived metrics at either individual or population level (Table 1). Instead, they observed a negative correlation between CBASS-derived ED50 values and classic experiment ∆Fv/Fm values at the individual level with the individual identified as most heat resistant in CBASS identified as the least resistant in the classic experiment.

For other response variables, correlations between CBASS and classic experiments were generally weak or even negative (Table 1). Klepac et al. (2024) found diverging trends between experiments in regards to all metrics evaluated at both the population and individual level. Voolstra et al. (2020) similarly reported minimal agreement between the two methods at both levels. It is also notable here that agreement at the population level for symbiont density and pigment variables was due to finding no differences between populations, which contradicts the agreement found in the Fv/Fm metrics that indicated one population was more resistant than the other. Only Evensen et al. (2021) observed strong agreement between methods for chlorophyll and protein concentrations, yet also reported minimal agreement when comparing changes in symbiont density and respiration rates.

#### Agreement between CBASS Fv/Fm metrics and other variables during experimental heat stress

CBASS-derived Fv/Fm metrics showed good agreement with classic Fv/Fm metrics, resolving similar patterns in individual and population-level heat resistance in three of the four studies (Table 2). With respect to other response variables, however, CBASS Fv/Fm did not correlate with any at the individual level (Table 2). At the population level, agreement was most often observed for chlorophyll concentrations and host protein content (Table 2). Notably, in Klepac et al. (2024), the population identified as being the most heat-resistant by the CBASS ED50 variable was identified as the least heat-resistant by all other CBASS and classic experiment variables. Similarly, in Voolstra et al. (2020), CBASS Fv/Fm resolved a significant difference between the two populations, whereas the classic experiment found no difference between populations across all four non-Fv/Fm response variables.

#### Agreement between CBASS and responses to in situ heat stress

Across the seven studies that compared CBASS results with in situ measurements, four relied on comparisons to previously published in situ data, while three directly evaluated CBASS outcomes against in situ measurements collected within the same study (Table S1). García et al. (2024) showed that CBASS ED50 positively correlated with ambient seawater temperature in one of the two coral species repeatedly tested throughout the year, which matches previous work showing potential summer priming effects (e.g. Brown et al. 2002). Of the three studies that collected in situ data, only two used the data to validate CBASS’s ability to predict in situ bleaching responses (McRae et al. 2024; Naugle et al. 2025). McRae et al. (2024) conducted CBASS assays with coral individuals across multiple sites and used photogrammetry before and after a marine heatwave to compare in situ bleaching responses with CBASS. They found that ED50 differences across sites generally matched in situ bleaching trends, but that CBASS bleaching responses were opposite those found in situ. In other words, CBASS Fv/Fm measurements matched in situ bleaching, but color-based bleaching scores did not. Importantly, the corals used to evaluate in situ bleaching were not the same individuals used in the CBASS assay and thus no colony-by-colony comparison can be made. Naugle et al. (2025) is the only study to directly compare the same colonies in CBASS and in situ. Contrary to McRae et al. (2024), they found that CBASS Fv/Fm measurements did not predict in situ heatwave survivorship. Instead, they found that the top 5% of individuals selected based on NDVI were 25% more likely to survive a heatwave compared to individuals at the median (Naugle et al. 2025). While the correlation was statistically significant, the R^2^ between NDVI and survivorship was very low (0.02–0.14), indicating that NDVI explained relatively little of the variation in heatwave survival. Overall, four of the studies reported mixed agreement between CBASS and in situ responses, two reported disagreement, and only one study found clear agreement (Supplementary Table 1).

## Discussion

### CBASS is primarily used by research organizations in a limited number of locations

Since its introduction in 2020, the application of CBASS has grown in published literature and spread in usage across the globe (Evensen et al. 2023a; Voolstra et al. 2024). The adoption of CBASS in scientific studies beyond universities and research institutes appears to be limited, potentially due to a lowered focus on publication. To date, coral bleaching experiments using CBASS have primarily focused on regions such as the Florida Reef Tract, the Red Sea, and the Great Barrier Reef, which represent major hotspots for coral collection (Fig. 2e). The overall number of studies is still relatively low and many coral reef regions have yet to be studied using the CBASS method, including the wider Caribbean, the Western Indian Ocean, and the biodiversity-rich Coral Triangle. This geographic bias generally aligns with similar trends for coral heat-stress experiments over the past thirty years and shows a failure to represent reefs in the Global South (McLachlan et al. 2020). At least in the restoration field, one limitation to the growth of CBASS in these areas may be barriers to funding. While data on coral restoration funding in the Global South is limited, some estimate that ~ 60% of funding in South-East Asia and ~ 75% in the Indian Ocean is under $50,000 USD per project (Hein and Staub 2021). Thus, even at a relatively affordable cost of $5000 USD (Evensen et al 2023b), CBASS systems may represent too high a price for smaller organizations. However, these results should be viewed in the context that the official CBASS framework was only published in mid-2023 (Evensen et al. 2023a), and geographic distribution of the methodology is likely to increase over time.

### CBASS experiments have similar taxonomic and depth bias as other coral bleaching experiments

Target species and taxonomic families used in CBASS experiments do not differ much compared to other heat stress experiments conducted in the previous thirty years, with acroporids, pocilloporids, and poritids being overrepresented (McLachlan et al. 2020) (Fig. 2a). This trend is also reflected in the distribution of coral morphologies, which is skewed toward branching species, likely due to their abundance, fast growth rates, relative ease of fragmentation, and role as important reef builders in the Indo-Pacific (Montaggioni 2005). While the focus on specific coral taxa accumulates detailed knowledge about target and abundant species, numerous coral species remain understudied (McLachlan et al. 2020). This trend may leave particularly large gaps in locations such as the Caribbean where non-branching groups such as *Orbicella* spp. are often the primary reef builders (Yranzo-Duque et al. 2025). Given the connection between coral biodiversity and overall reef resilience, identifying thermally tolerant corals from diverse taxa has important implications for conservation and restoration efforts globally.

Coral collections for CBASS experiments have been limited to depths of up to 16.4 m, indicating a strong bias toward shallow reefs. However, this may partially be a result of commonly conducting specimen collection and assays on the same day, and/or limited bottom times at depth. Additionally, given that the main coral bleaching triggers are high temperatures and irradiance, or their combination (Helgoe et al. 2024), shallow-reef corals are often most impacted by coral bleaching and thus represent obvious targets when assessing thermal thresholds. Unfortunately, with research suggesting that deeper reefs provide limited refuge from mass bleaching (e.g., Frade et al. 2018), the omission of mesophotic corals from these experiments likely reduces our ability to understand how thermal tolerances change across depth.

The majority of sampled corals were collected from the wild, whereas others originated from coral nurseries. While nurseries have the advantage that the genetic identity of corals and symbiont type is often known (e.g., Evensen et al. 2021; DeMerlis 2024; Stankiewicz et al. 2025), corals may be acclimatized to localized environments (e.g., Klepac et al. 2024), which could potentially influence their performance in CBASS experiments. However, including both origin types in CBASS experiments enables a more comprehensive understanding of coral responses across different ecological contexts.

### Most CBASS experiments have good biological replication but suffer from inadequate tank replication

CBASS experiments generally followed suggested frameworks for genotypic replication but likely failed to capture intra-colony variation and often suffered from pseudo-replication due to a lack of tank replication. Aside from studies with aquarium-reared specimens (Alderdice et al. 2022; Dörr et al. 2023; Schlotheuber et al. 2024) where authors acknowledged the limited biological replication and risks of pseudo-replication (e.g., Schlotheuber et al. 2024), most CBASS experiments had high genetic replication using a median of 9.5 colonies. Genetic determination is the most reliable method for confirming the uniqueness of genets, but it is recognized that genetic identification is not always feasible due to cost (Grottoli et al. 2021). As such, sampling colonies at a specific distance apart is often used as a proxy to ensure independent genets are collected (e.g., Voolstra et al. 2020, 2021; Naugle et al. 2021). Distance between colonies was frequently reported in CBASS experiments (34%), with many accepting a minimum distance of five meters between colonies to reduce the chance of sampling clones, often citing Baums et al. (2006). Thus, it appears that, aside from using genetic markers or colony fusion (Blanco-Pimentel et al. 2024), this sampling approach is widely accepted across studies and contributes to the standardization of CBASS experiments. Regardless, one should be aware that additional factors such as morphological plasticity (e.g., Todd 2008; Flot et al. 2011), long-distance clonal dispersal (e.g., Gélin et al. 2017), and cryptic species (e.g., Johnston et al. 2022) could complicate sampling.

Most CBASS experiments used one fragment (ramet) per parent colony (genet) per tank, indicating that the majority of experiments may not capture inherent biological variation within colonies (Grottoli et al. 2021). Fragment size was reported in over half of CBASS studies, whereas it is rarely reported in other coral bleaching experiments (McLachlan et al. 2021) and has not been incorporated into experimental guidelines (Grottoli et al. 2021). With evidence suggesting that fragment size influences coral stress responses and varies between species and measured response variables (Shenkar et al. 2005; Pausch et al. 2018; Nielsen et al. 2022), the comparatively high reporting of fragment sizes in CBASS experiments can be considered a positive development and should be encouraged. The differences in reported size units likely stem from variations in coral morphology within experiments, as length—the most commonly reported size metric—coincides with the dominance of branching morphologies.

Notably, more than half of CBASS experiments suffer from pseudo-replication at the level of the experimental unit because they did not replicate experimental tanks. This makes it impossible to distinguish treatment effects from potential tank effects and interferes with independence of biological replicates (Hulbert 1984). It is crucial to mitigate this issue by implementing multiple independent experimental units, as has been recommended for ocean acidification experiments (Cornwall and Hurd 2016). Recommended minimum tanks per treatment vary, but two (Grottoli et al. 2021) or three (Underwood 1997; Cornwall and Hurd 2016) are likely sufficient. However, the base design of CBASS inherently limits tank replication, as most systems have two prefabricated controllers that can only connect to four tanks at most. As such, researchers are forced to choose between a design involving four treatments and two tank replicates (e.g., Voolstra et al. 2020; Evensen et al. 2021) or eight treatments with one tank replicate (e.g., Klepac et al 2024; Cunning et al 2024). To circumvent the eight-treatment design issue, some studies repeated the assay at least twice with random tank-treatment assignment (e.g., Klepac et al 2024).

### Few CBASS experiments have acclimation and recovery phases after coral collection and fragmentation

Acclimation prior to a heat stress test is an important aspect in classic experiments (Grottoli et al. 2021) to allow for recovery from collection and handling stress, healing after fragmentation, and acclimation to new tank and light conditions, but this was often not considered in CBASS experiments. Nevertheless, we found that an acclimation period prior to the CBASS experiment was sometimes conducted for several reasons, even though this is not recommended in the original paper (Evensen et al. 2023b). For example, short-term acclimation aimed to prime specimens to experimental baseline conditions (Naugle et al. 2021; Delgadillo-Ordoñez et al. 2024; Stankiewicz et al. 2025). In contrast, long-term acclimation was used in transplantation experiments (Klepac and Barshis 2020, 2022) to acclimate specimens to specific light levels (Lane 2023; Ducret et al. 2024), specific temperature conditions (DeMerlis 2024), or following transfer to a nursery site (Alderdice et al. 2024). Studies primarily stated recovery periods after fragmentation without providing detailed explanations (e.g., Cunning et al. 2021; Cornwell et al. 2021; Denis et al. 2024). These periods likely served logistical purposes (e.g., Brown et al. 2024) but are more importantly used to distinguish experimental effects from fragmentation-induced stress (Lock et al. 2022) or habitat preconditioning (e.g., Denis et al. 2024). As such, while acclimation periods extend the otherwise fast CBASS experiment, they are beneficial in controlling for potentially confounding handling, fragmentation and differential habitat stressors, ensuring a standardized baseline for comparisons. In regard to fragmentation, acclimation could be particularly important for coral species with non-branching morphologies as cutting with band saws or other means of fragmentation leads to large areas of exposed edges and often intense mucus production.

### Temperature treatments are standardized and well-justified whereas light levels are not

The original CBASS design recommends that appropriate settings of temperature and light should vary across experiments due to differences in season, location, species studied, and system modifications (Evensen et al. 2023b). This was especially reflected in the choices made for control and treatment temperatures. Most studies justified their choice of control temperature based on the study location’s MMM as the baseline, but some studies included an additional ambient temperature treatment to account for potential differences between the MMM-based baseline and the ambient temperature at the time of the experiment.

Treatment temperatures varied widely, primarily due to differences in thermal tolerances between species and populations. Some experiments increased overall treatment temperatures to better assess differences between treatments and elicit stronger coral responses (Brown et al. 2024), while others decreased the highest treatments due to near complete mortality (Stankiewicz et al. 2025). Given the variation in appropriate temperature treatments for individual species and environments, Evensen et al. (2023b) recommended that CBASS users perform pilot experiments to determine the temperatures that elicit an appropriate range of bleaching responses (e.g., Klepac and Barshis 2020; Savary et al. 2021; Brown et al. 2024), enabling accurate dose–response modeling (Voolstra et al. 2024). Justifications for the chosen treatment temperatures were less frequently provided compared to those for baseline temperatures, with most supporting treatments based on the original experimental design (e.g., McRae et al. 2024) or ensuring a sufficiently broad bleaching response (e.g., Savary et al. 2021). Given that standardization is a key goal of CBASS, the absence of explicit justifications can reduce inter-experiment comparability, and clearly reporting these decisions should thus be encouraged to improve transparency.

Temperature profiles turned out to be highly standardized, with ramp-up and heat-hold being 3-h long across all studies. However, the consistent ramp-up duration mixed with variation in temperature treatments inevitably leads to differing temperature ramp rates between experiments (°C h^−1^) (Grottoli et al. 2021). While this may be acceptable for short-term assays given that consistent ramping and heating durations are proposed to facilitate comparability (Grottoli et al. 2021), heating rate remains an important factor to consider, as more rapid temperature increases could induce stronger stress responses (e.g., Middlebrook et al. 2010; Sahin et al. 2023).

In stark contrast to temperature, there was a lack of standardization of light intensity throughout studies, which used light levels ranging from 22 to 1600 μmol photons m^−2^ s^−1^. Light intensity, spectral quality (i.e., the distribution of wavelengths), and the justifications for the levels used are crucial aspects of coral heat stress experiments, as light can act as a bleaching trigger both independently and in combination with temperature (Brown 1997; Helgoe et al. 2024). It is noted that some of the variation in chosen light intensities was due to studies incorporating light as an additional treatment factor (Lane 2023; Ducret et al. 2024), differences in culturing conditions (Dörr et al. 2023), or efforts to match in situ measurements (Voolstra et al. 2021; McRae et al. 2024). Additionally, it is encouraging that, despite the wide range of light intensities reported across studies, the mean and median light intensities across experiments generally fell within the recommended range (250–500 μmol photons m^−2^ s^−1^), which is suggested for stimulating maximal photosynthesis, avoiding photodamage, and matching natural environments, as proposed in bleaching experiment guidelines (Grottoli et al. 2021). Regardless, standardization of light levels is essential for an assay that aims to standardize short-term stress tests, especially considering that Fv/Fm, the most commonly used metric to determine bleaching phenotype in CBASS, is strongly influenced by light history (Castro-Sanguino et al. 2024) and instrument settings (see below).

### Chlorophyll *a* fluorescence is measured in most CBASS experiments but standardization and reporting can be improved

Most studies measured chlorophyll *a* fluorescence using PAM fluorometry in dark-adapted states (Fv/Fm), indicating a high degree of comparability across experiments, as it is a widely reported metric of coral health (Warner et al. 2010; Bhagooli et al. 2021). Notably, all studies that measured chlorophyll *a* fluorescence used PAM instruments, minimizing differences due to instrument variability. Since all reported instruments were manufactured by Walz GmbH and operate on the same pulse-amplitude modulation (PAM) principle, measurement consistency is relatively high. This is advantageous because differences between single-turnover instruments (such as the PAM) and multiple-turnover instruments have been observed, which could otherwise complicate comparisons (Warner et al. 2010). Furthermore, the use of Fv/Fm-derived ED50 values was highly consistent across studies, indicating strong comparability. Values were derived from dose–response models based on three-parameter log-logistic regressions (e.gCunning et al. 2021; Voolstra et al. 2021; Evensen et al. 2022), suggesting high standardization. Two studies derived ED25 values in experiments that did not lead to a decline of Fv/Fm by 50% as it was a more appropriate and robust parameter in these cases (Evensen et al. 2023a; Klepac et al. 2024).

Despite this consistency, standardization can be improved further and remains essential across studies. Seasonal and diel fluctuations in photosynthetic efficiency occur naturally (Brown et al. 1999; Hoegh-Guldberg and Jones 1999; Gorbunov et al. 2001; Warner et al. 2002), often due to reversible photoinhibition (Fitt et al. 2001; Gorbunov et al. 2001). Measurement timing after sundown and a dark-adaptation period of mostly 60 min were consistent across CBASS experiments, indicating a high degree of standardization and enhancing comparability across studies. Only a few deviations were reported (Marzonie et al. 2022; Naugle et al. 2024; Denis et al. 2024). Sufficient dark adaptation ensures that all photosystem II (PSII) reaction centers are in an open state and that non-photochemical quenching is minimized (Walz 2023). However, extended periods in the dark can reduce the PSII acceptor pool through chlororespiration, potentially lowering maximal quantum yield (Warner et al. 2010; Walz 2023). This issue can be mitigated by briefly pre-illuminating the sample with far-red light before taking dark-adapted measurements (Hill and Ralph 2008; Warner et al. 2010; Walz 2023).

Instrument settings also play a crucial role in fluorescence measurements, as they directly influence recorded values. For example, an actinic effect, i.e., the occurrence of photochemical reactions, should be avoided by considering the measuring light intensity since this would artificially increase F0 (Warner et al. 2010). Although PAM settings are reported in at least half of the studies, inconsistencies remain in which parameters are documented, even though all instruments share the same general settings and only differ in their adjustable ranges. In addition to instrument settings, one should consider measurement geometry, as the distance and angle of fiber-optic cables influence how light interacts with the coral tissue, affecting fluorescence readings. Coral skeleton and tissue structure modulate the internal light environment for symbionts (e.g., Kühl et al. 1995; Enríquez et al. 2005; Wangpraseurt et al. 2012), which can, in turn, affect PAM measurements (Wangpraseurt et al. 2019).

Although Fv/Fm appears to be a standardized and commonly used response variable, it is insufficient to reveal symbiotic breakdown on its own (e.g., Middlebrook et al. 2010), as it cannot be used as a substitute for direct photosynthesis measurements using respirometry (Hoegh-Guldberg and Jones 1999; Lesser and Gorbunov 2001; Warner et al. 2010; McLachlan et al. 2020) and does not directly quantify bleaching (i.e., the loss of symbionts and/or photosynthetic pigments). Notably, reductions in Fv/Fm can result solely from increased light exposure within the tissue during bleaching and thus do not always accurately reflect the loss of photosynthetic performance or the degree of pigment or symbiont loss (Gomez-Campo and Baums 2025). In addition, discrepancies between the light regimes a coral is acclimatized to and experimental light levels (and quality) can impact photochemical but not visual bleaching responses (Castro-Sanguino et al. 2024; Gomez-Campo and Baums 2025). Seasonality also likely impacts CBASS ED50 with one study showing higher ED50 values in summer (García et al. 2024). It is also concerning that Fv/Fm ED50 only resolved similar rankings in thermal tolerance in two of the three studies that tested CBASS reproducibility (Cunning et al. 2021, 2024). Fv/Fm is therefore not a simple predictor of thermal sensitivity during acute heat-stress, with bleaching phenotype response variables (see next section) representing a more reliable measure for bleaching severity (McLachlan et al. 2020). 

### Coral host responses are underrepresented compared to measurements of bleaching phenotype

Common analyses to assess bleaching phenotype included the quantification of chlorophyll pigments, symbiont cell density counts or image analysis to measure paling. However, 31% of studies did not measure any of these response variables, which is an even higher percentage than shown for other types of coral bleaching experiments (McLachlan et al. 2020). While image analyses can be a simple and cost-effective tool to quantify bleaching (Johnsen 2016), they have the inherent limitation that they often rely on the subjective interpretation of humans. Non-invasive, reflectance-based measurements, such as NDVI as a proxy for chlorophyll *a*, are a compelling approach by removing observer bias through objective measurements and providing spectral resolution. Naugle et al. (2025) supports the predictive ability of NDVI to identify resilient coral genotypes, but the fact that the R^2^ values in this study are low (i.e., 0.02–0.14) indicates that much of the variation in survivorship was unaccounted for. However, given that some of the survival surveys were conducted seven months after the bleaching event, it is possible that disease and predation following bleaching were a major contributor to mortality of survivors (e.g. Brandt and McManus 2009; Miller et al. 2009; Bruckner et al. 2017), resulting in lowered R^2^ values. Regardless, while reflectance-based measurements in coral reef ecophysiology remain limited to date and standardization needs improvement, reflectance-based NDVI offers a promising non-invasive technique in addition to existing methods to assess the physiological condition of corals (Gomez-Campo and Baums 2025; Watty et al. 2024, 2026).

Measurements of the holobiont phenotype are less frequently reported among CBASS experiments, with host protein being the most commonly measured variable. Overall, the coral host response is underrepresented, likely due to the difficult implementation of host response measurements in CBASS experiments. For example, common metrics, such as growth rate and calcification, require more time to measure and are more suited for long-term experiments (Grottoli et al. 2021). Even measurements of photosynthesis and respiration are difficult to conduct over such short timescales, and studies have resorted to running separate rapid heat-stress assays (4–5 days) using new sets of ramets from the same colonies due to the short duration of CBASS (e.g., Evensen et al. 2023a).

The omission of coral host responses and non-Fv/Fm-derived bleaching metrics in 31% of studies is important as these metrics alone are likely insufficient to identify thermally resistant genotypes and/or populations. While Voolstra et al. (2020) used correlations between CBASS and classic experiment Fv/Fm responses to argue that they are a reliable indicator of heat stress resilience, but Klepac et al. (2024) showed that the genotype with the highest CBASS-derived ED50 exhibited the poorest outcomes in all other variables in both the CBASS and classic experiment. Naugle et al. (2025) also showed that Fv/Fm-derived metrics failed to predict in situ marine heatwave survivorship. This highlights that a complementary second, or single more robust, variable is necessary to get a more accurate estimate of heat stress resistance (see also Gomez-Campo and Baums 2025).

### Frequent tissue sloughing and mortality require rethinking of the highest temperature treatment

Typical CBASS temperature profiles include a very high temperature treatment (MMM + 9 °C) that is necessary to elicit a broad bleaching response and drive response variables to zero, as a prerequisite of the typically used three parameter dose–response curves for ED50 estimation (Ritz and Streibig 2005; Knezevic et al. 2007). However, these extreme exposures come with significant drawbacks as they effectively represent heat shock treatments that can lead to mortality, tissue sloughing, or necrosis, as reported in several CBASS studies (Voolstra et al. 2020; Evensen et al. 2023a; Lane 2023; DeMerlis 2024; Schlotheuber et al. 2024). These extreme temperatures were ecologically relevant in the acute stress tests that predated CBASS (e.g. Palumbi et al. 2014; Morikawa and Palumbi 2019) as corals used in those studies came from back-reef pools that experience rapid heating over tidal cycles but are problematic for corals from most other habitats. Tissue sloughing and necrosis pose a particular issue for Fv/Fm measurements, as it becomes inappropriate to use a PAM fluorometer when no tissue remains. If no symbiont-related photosystem II reaction centers are present, photosynthetic capacity cannot be quantified, and the Fv/Fm value is effectively missing. It appears that some studies reporting ‘0’ values in their highest treatments may have treated these missing values as true zero readings (e.g., Klepac et al. 2024, 37 °C treatment in Fig. 2; Evensen et al. 2022, 39 °C treatment in Fig. 2), potentially leading to incorrect group averages and sample sizes. Tissue sloughing and necrosis can also introduce confounding factors when measuring physiological responses by impacting water quality, leading to an adverse effect on the health of other fragments within a tank, particularly when only one tank per treatment is used.

### More research is needed to determine whether corals respond similarly to acute and chronic heat exposures

While the short duration of CBASS experiments is an attractive logistical advantage, the crucial question is whether coral responses to acute heat stress predict responses to longer heat exposures. Our assessment of the four method comparison studies and two in situ studies suggests that it is at present not possible to conclusively answer this question. On one hand, CBASS shows some capacity to distinguish long-term thermal tolerance differences between top and bottom performers, and at the population and site level. Voolstra et al. (2020) found that CBASS resolved similar site differences as a classic experiment, Cunning et al. (2024) showed that both approaches identified the same genotypes as most and least thermally resistant, and Naugle et al. (2025) found that CBASS-derived NDVI metrics generally predicted top performers following an in situ bleaching event. These results suggest CBASS may capture ecologically meaningful variation across broad groupings. On the other hand, differences in experimental design limit the ability to compare the responses in acute vs classic experiments. For example, light levels differed substantially between CBASS and classic experiments in the original study that introduced CBASS, potentially confounding temperature effects (Voolstra et al. 2020). Furthermore, while some studies show significant correlations between CBASS and classic experiments, spatial autocorrelation may inflate statistical significance while small sample sizes preclude robust within-site level analysis (e.g., Voolstra et al. 2020; Klepac et al. 2024). Secondly, while alignment between experiments is generally strong when using Fv/Fm-derived metrics, this is not the case for nearly every other response metric used (Tables 1, 2). Interestingly, the McRae et al. (2024) in situ data supports this observation, showing that CBASS ED50 correlated with in situ bleaching, but color-based bleaching metrics did not. It is perhaps not surprising that metrics such as chlorophyll and host protein concentrations can show large differences between CBASS and longer heat-stress tests, as declines are often delayed compared to Fv/Fm (Klepac et al. 2024). Naugle et al. (2025) showed that even when there is a significant relationship between non-Fv/Fm CBASS metrics, such as NDVI, and in situ heatwave survival, the relationship is weak explaining only 2–14% of the variation in survivorship. Disregarding impacts of post-bleaching disease and predation, whether this constitutes a useful predictor likely depends on the context and intended application. Other studies that compared CBASS results to previous literature or in situ Fv/Fm data demonstrated a range of weak to strong support for CBASS (Supplementary Table 1). In general, these lines of evidence suggest that there is likely a significant difference in how corals respond to acute and chronic heat stress, supporting ecological theory and genetic work (Rezende et al. 2014; Savary et al. 2021; Humanes et al 2024). This raises questions regarding the utility of CBASS-based thermal tolerance rankings to inform thermal tolerance research and restoration decisions and highlights that more research is needed to test whether coral responses to acute heat stress are representative of responses to chronic heat exposures across habitats and environmental conditions.

## Conclusion and recommendations

In conclusion, this review serves as an early assessment of the application and utility of CBASS as a standardized tool for rapid coral thermal tolerance assessment, which is gaining traction among diverse user groups. By providing this preliminary evaluation, we aimed to highlight strengths of this new tool as well as identify areas for optimization and standardization in methodology and reporting, building on existing frameworks for coral bleaching experiments to enhance comparability and reproducibility (Grottoli et al. 2021; McLachlan et al. 2020), while drawing attention to potential knowledge gaps and limitations. To improve the reliability, comparability, and ecological relevance of CBASS experiments, we provide the following seven recommendations:

### Recommendation 1 – Increasing taxonomic, depth, and geographical representation

While we recognize that CBASS is still relatively new, we encourage users to include more species and a broader depth range to address the current bias in shallow-water branching corals. Knowledge gained from this expansion will diversify coral bleaching research and facilitate reef restoration and conservation, particularly in areas where non-branching corals are the primary reef builders. Additionally, reducing system costs, e.g., by using cheaper tanks or alternative cooling mechanisms (Reuter et al. 2025), could further expand its use in the understudied Global South and among restoration groups.

### Recommendation 2 – Avoiding pseudo-replication through adequate tank replication

As stated above, replication designs may vary, but two (Grottoli et al. 2021), or three (Underwood 1997; Cornwall and Hurd 2016) tanks are likely sufficient for CBASS. For designs with more than four treatments, the experiment may be run in multiple rounds to increase replication, or multiple CBASS set-ups could be used. If repeated rounds are run, care must be taken to avoid temporal autocorrelation. One tank of each temperature should be included in each iteration of the entire CBASS run. However, non-uniform acclimatization times across rounds become an unavoidable, though likely lesser, problem. Each tank should contain at least two fragments per parent colony to capture potential intra-colony variation. Extra care is recommended when using an acrylic wall to divide a tank to increase the number of experimental compartments (e.g., Evensen et al. 2023b), as compartments within the same tank may share environmental conditions. True replication is best achieved with separate tanks whenever possible (Cornwall and Hurd 2016).

### Recommendation 3 – Implementing a recovery phase to minimize handling and fragmentation stress

An acclimation and recovery phase before the start of a CBASS experiment can help ensure standardized baselines for cross-experiment comparisons and reduce the potential for fragmentation and handling stress to confound responses to heat stress. The recovery phase may be especially important for non-branching species where sampling and/or fragmentation methods result in exposed edges, tissue damage, and mucus production.

### Recommendation 4 – Measuring in situ light levels to inform experimental light regimes

Given the importance of light levels, particularly in the context of Fv/Fm-derived tolerance metrics, we recommend that experimental light levels should reflect in situ conditions at the respective collection depth, and that in situ light intensities be reported alongside experimental light levels. If corals from locations with different light conditions are used, we suggest using intermediate levels or the lower light intensity as sudden increases are more stressful than sudden decreases (Osinga et al. 2008). An acclimation period might be optimal to ensure both populations are acclimatized to the same experimental conditions as low light levels could otherwise artificially benefit corals from the higher-light environment when assessed for their heat tolerance.

### Recommendation 5 – Reporting fluorometer settings and measuring more responses than Fv/Fm

PAM instrument settings, dark-acclimation time, and measurement geometry should be reported, as they influence Fv/Fm readings. Additionally, given the limitations of Fv/Fm in providing an overall picture of the coral holobiont health (Gomez-Campo and Baums 2025), we suggest using at least one additional response variable, such as chlorophyll, protein content, or NDVI, alongside Fv/Fm measurements to quantify the bleaching phenotype.

### Recommendation 6 – Adjusting maximum temperatures to avoid tissue sloughing and mortality

Extreme high temperatures that have the potential to cause sloughing or necrosis of tissue should be avoided to ensure response measurements reflect physiologically meaningful bleaching responses rather than severe physiological breakdown. This is especially important for PAM readings, as one cannot replace a NA reading with the value 0 in statistical analyses and models. Pilot studies can be used to determine an acceptable maximum temperature, and increased flow rates can help reduce the effects of decreased water quality from tissue degradation of less resistant genotypes.

### Recommendation 7 – Conduct more research to evaluate if responses to acute heat stress reflect those to longer-term marine heatwaves

CBASS offers many advantages, including low cost and quick pace, but its limitations, such as the use of extreme and rapid temperature treatments make it important to contextualize results appropriately. Specifically, the current assessment of the few studies comparing CBASS to longer heat exposures highlights that there is mixed agreement between acute and classic experimental approaches (Voolstra et al. 2020; Evensen et al. 2021; Klepac et al. 2024), Fv/Fm-derived responses and other bleaching metrics (Voolstra et al. 2020; Evensen et al. 2021; Klepac et al. 2024), and CBASS-derived and in situ bleaching responses (McRae et al. 2024; Naugle et al. 2025). As such, it appears that the most supported use case of CBASS is to distinguish acute stress tolerance between broad groups such as across sites and/or between the top and bottom performers. However given the limited agreement described above, it remains unclear whether responses to acute heat stress truly represent those of longer-term heat stress. We urge the community to conduct more research and compare CBASS outcomes with in situ bleaching responses and heat stress experiments that more closely mimic the natural environment. Until then, we caution relying on CBASS alone to inform coral bleaching research, reef conservation and the selection of genotypes for restoration planning.

## Supplementary Information

Below is the link to the electronic supplementary material.

Supplementary file1 (DOCX 26 KB)

Supplementary file2 (XLSX 55 KB)

## Data Availability

The data used in this manuscript came directly from the studies that were included in the review. Github repositories for original studies were used when correlational data were not apparent from the study itself.
