# Supplementary material for: Acute heat-stress testing in coral bleaching research: a review of research using the Coral Bleaching Automated Stress System (CBASS)
Source: Coral Reefs. 2026 Apr 4;45(4):2059–81. doi: 10.1007/s00338-026-02862-7 (PMC13490275; doi:10.1007/s00338-026-02862-7)
Supplement: Supplementary file 1 [file 338_2026_2862_MOESM1_ESM.docx]

**Supplementary Information of the Manuscript “Acute heat-stress testing in coral bleaching research: A review of research using the Coral Bleaching Automated Stress System (CBASS)”**

**Author Information:**

Ilan E. Bubb^1,2,*^, Kay Watty^1,*^, Lyza Johnston^2^, Verena Schoepf^1^

^1^Department of Freshwater and Marine Ecology, Institute for Biodiversity and Ecosystem Dynamics, University of Amsterdam, Amsterdam, The Netherlands

^2^Johnston Applied Marine Sciences, Saipan, Commonwealth of the Northern Mariana Islands, United States of America

* corresponding authors:

Ilan Bubb; ibubb@jamssaipan.com

Kay Watty; khw@watty.eu

**Supplementary Figures:**

**Table S1** Comparison of CBASS results to in-situ data. CBASS was either compared to previous literature or to data obtained in the same study. Type of comparison indicates whether CBASS was compared to previous literature or in situ surveys. The rating provides an indication of whether in situ data supported CBASS results, went against them, or were mixed.

| **Study** | **Type of comparison** | **Cited literature** | **Type of in situ data** | **Rating** | **Details** |
| --- | --- | --- | --- | --- | --- |
| Brown et al. 2024 | Indirect | Ainsworth et al. 2021; Brown et al. 2023a | bleaching prevalence (% bleached); benthic community cover change; symbiont density; photosynthetic efficiency | against | Corals from variable temperature sites showed higher thermal thresholds but the highest mortality during the 2020 marine heatwave |
| Evensen et al. 2022 | Indirect | Osman et al. 2018; Monroe et al. 2018; Anton et al. 2020 | bleaching severity in historical records; bleaching surveys (% bleached) | mixed | While thermal thresholds relative to local MMM support historical bleaching observations, absolute thresholds suggest opposite trends depending on the species |
| Garc**í**a et al. 2024 | Direct | - | in situ photosynthetic efficiency (Fv/Fm) | mixed | One species showed a negative correlation between in situ Fv/Fm and CBASS ED50 and a positive correlation between ED50 and seawater temperature; the other species showed no correlations |
| McRae et al. 2024 | Direct | - | bleaching scores of subsequent bleaching event in the same year | mixed | Fv/Fm and ED50 matched in situ bleaching trends while CBASS bleaching scores did not |
| Naugle et al. 2025 | Direct | - | Image analysis (pigmentation scores); percent mortality | mixed | Retained NDVI predicted in situ bleaching better than photosynthetic efficiency (Fv/Fm) |
| Voolstra et al 2020 | Indirect | Pineda et al. 2013 | bleaching surveys (% dead vs. live colonies) | support | Experimental Fv/Fm decline matched higher in situ bleaching during a previous bleaching event |
| Voolstra et al 2021 | Indirect | Osman et al. 2018 | bleaching severity in historical records | against | ED50 were found to be lowest in the northern Red Sea while corals in this region bleached less in the past |
